# Platelet-derived microvesicles isolated from type-2 diabetes mellitus patients harbour an altered miRNA signature and drive MDA-MB-231 triple-negative breast cancer cell invasion

**DOI:** 10.1371/journal.pone.0304870

**Published:** 2024-06-20

**Authors:** Anca Tutuianu, Chinedu A. Anene, Mikayla Shelton, Valerie Speirs, Donald C. Whitelaw, Joanne Thorpe, Wayne Roberts, James R. Boyne

**Affiliations:** 1 School of Applied Sciences, University of Huddersfield, Huddersfield, United Kingdom; 2 Biomedical Science, School of Health, Leeds Beckett University, Leeds, United Kingdom; 3 Institute of Medical Science, School of Medicine, Medical Sciences and Nutrition, University of Aberdeen, Aberdeen, Scotland; 4 Department of Diabetes and Endocrinology, Bradford Royal Infirmary, Bradford, United Kingdom; Rutgers University, UNITED STATES

## Abstract

The underlying causes of breast cancer are diverse, however, there is a striking association between type 2 diabetes and poor patient outcomes. Platelet activation is a common feature of both type 2 diabetes and breast cancer and has been implicated in tumourigenesis through a multitude of pathways. Here transcriptomic analysis of type 2 diabetes patient-derived platelet microvesicles revealed an altered miRNA signature compared with normoglycaemic control patients. Interestingly, interrogation of these data identifies a shift towards an oncogenic signature in type 2 diabetes-derived platelet microvesicles, with increased levels of miRNAs implicated in breast cancer progression and poor prognosis. Functional studies demonstrate that platelet microvesicles isolated from type 2 diabetes patient blood are internalised by triple-negative breast cancer cells *in vitro*, and that co-incubation with type 2 diabetes patient-derived platelet microvesicles led to significantly increased expression of epithelial to mesenchymal transition markers and triple-negative breast cancer cell invasion compared with platelet microvesicles from healthy volunteers. Together, these data suggest that circulating PMVs in type 2 diabetes patients may contribute to the progression of triple-negative breast cancer.

## Introduction

There is a profound association between breast cancer (BC) and type 2 diabetes mellitus (T2DM; diabetes), with diabetic women being more likely to both develop BC [1–3] and die from the disease [4, 5]. The mechanisms underpinning this association remain poorly understood. however, platelet activation is a common feature of both diabetes [6] and BC [7, 8] and is driven by metabolic reprogramming, a central hallmark of both pathologies [9]. Indeed, platelet activation is linked to disease progression and poor patient outcomes for a range of cancers [10–14] and has been reported to drive tumourigenesis via a multitude of pathways [15], including enhancing growth and immune invasion [16].

Platelet-derived microvesicles (PMVs) are emerging as key effectors in the aetiology of cardiovascular disease and cancer [17]. PMVs are released from platelets and differ from the widely studied small extracellular vesicles (also known as exosomes) in both their size and their genesis [18]. In addition to their role in thrombosis [19], PMVs also act as reservoirs for bioactive molecules derived from their parent cells [20], with platelet-derived micro-RNAs (miRNAs) attracting significant interest [21, 22]. Underpinning this is a growing body of evidence demonstrating that PMVs deliver miRNAs to a range of cells leading to altered transcriptomes and target cell phenotypes [23–26].

It is becoming increasingly apparent that T2DM induces its own miRNA signature [27–30]. For example, the expression profile of five plasma microRNAs; miR-15a, miR28-3p, miR-126, miR-223, and miR-320, can be used to distinguish T2DM patients from healthy controls [31]. Other groups have highlighted that miR-223 and miR-146a expression is altered in diabetic patient plasma and platelets [27] and there is evidence that altered miRNA expression is associated with poorly controlled T2DM [32]. This raises the intriguing possibility that a T2DM-derived PMV miRNA signature might contribute to the increased risk of breast cancer progression in diabetic patients. Indeed, several studies lend support to this model, including pioneering work establishing that small extracellular vesicles in obesity and metabolic disease are important drivers of cancer progression for several cancer types [33–36]. Moreover, and of particularly relevance to this study, is work reporting that exosomes from diabetic adipocytes and human mesenchymal stem cells drive progression in breast cancer and that plasma exosomes of patients with T2DM carry a distinct profile of miRNAs of known significance for prostate cancer progression that contribute functional effects in a range of cancer models [37].

Here small-RNA sequencing of T2DM- and normoglycaemic-derived PMVs revealed significant differences in the abundance of numerous BC-associated miRNAs. Bioinformatic analysis of these data revealed a shift in T2DM-derived PMVs towards increased expression of pro-tumorigenic miRNA and reduced expression of miRNA reported to function as BC tumour suppressors. To investigate the impact of diabetic PMVs compared with normoglycaemic control PMVs on breast cancer cells, co-incubation experiments were performed. Strikingly, we observed significant increases in cell invasion index and key epithelial-mesenchymal transition (EMT) genes in triple-negative breast cancer (TNBC) cells co-incubated with diabetic PMVs compared with TNBC co-incubated with normoglycaemic PMV controls. Moreover, TNBC cells co-incubated with T2DM-dervied PMVs exhibited increased levels of miRNAs identified as enriched in diabetic samples, suggesting that PMVs may induce these phenotypic changes in BC cells via delivery of differential profiles of miRNAs.

## Results

### T2DM-derived PMVs harbour a distinct miRNA profile

**Fig 1:**
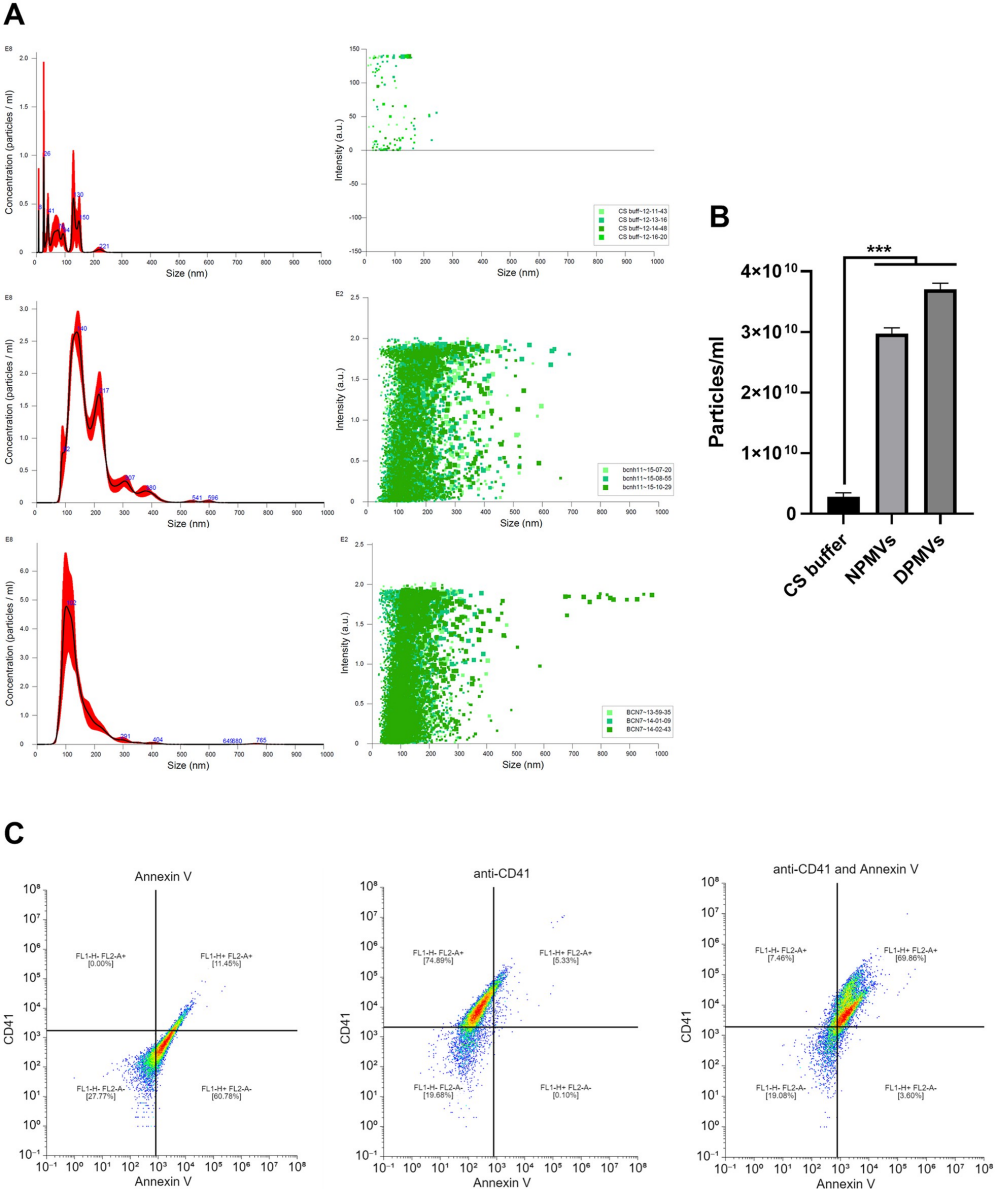
Isolation and characterisation of diabetic PMVs. (A) Analysis of Tyrode’s buffer, healthy control PMVs (NPMV) and diabetic-patient derived PMVs (DPMV) by NTA. Left panels display particle concentrations (1x10^8^) and size, right panels display individual particle size verses intensity (a.u.), n = 3. (B) PMV particle concentration for Tyrode’s buffer, NPMV and DPMV, n = 3. (C) PMVs were labelled with Annexin V-FITC and/or CD41a-PE, gated around singlets and 10,000 events collected. Example dot plot for single and dual labelled PMVs with quadrant analysis, n = 3. Data are representative of all PMV samples, *** = p<0.001.

**Fig 2:**
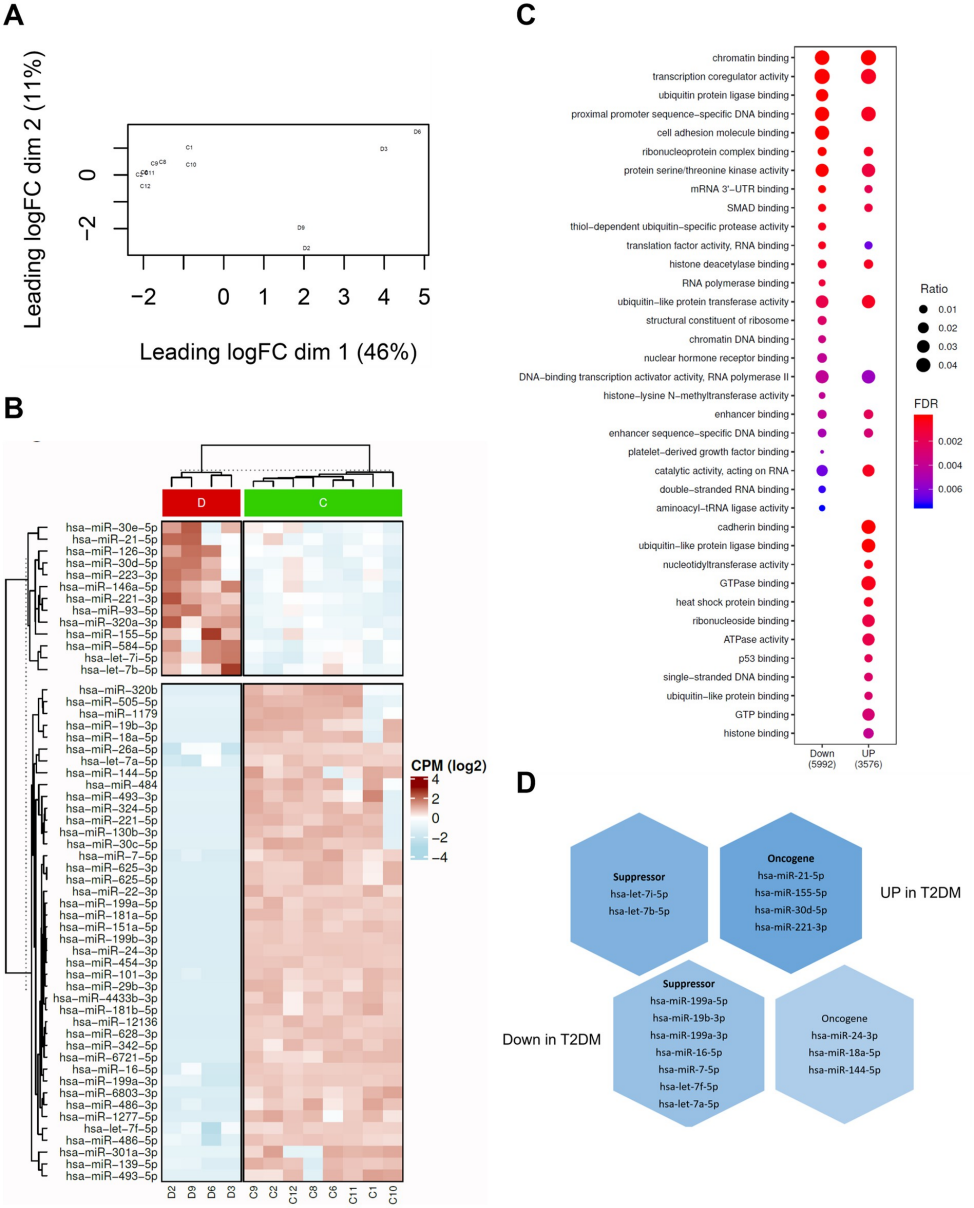
PMVs isolated from poorly controlled diabetic patients harbour an oncogene-polarised miRNA profile compared with PMVs derived from healthy volunteers. (A) Multi-dimensional scaling (MDS) plot of miRNA profiles in diabetic PMV samples (D; n = 4) and healthy controls (C; n = 8). (B) Heatmap of expression profiles of all differentially expressed miRNAs in diabetic PMVs compared with normal PMVs. Within the plot, red colour shows diabetic PMVs and green normal. (C) Cluster plot of enriched biological processes associated with mRNA targets of the down regulated (Down) or up regulated (Up) miRNAs. Gene ratio—genes in ontology/total gene set and false discovery rate (FDR)-. mRNA transcripts represented in the plot are experimentally validated miRNA-mRNA interactions from the miRTarBase database. (D) Distribution of known tumour suppressor and oncogenic miRNAs in the set of differentially expressed miRNAs in diabetic PMVs compared with normal PMVs.

There is emerging evidence that diabetic patients harbour altered circulating miRNA profiles and that these may have utility as biomarker panels [38]. To investigate if PMVs isolated from poorly controlled T2DM patients possess distinct miRNA cargo human blood was taken by venepuncture from T2DM patients with HbA1C>50mmol/mol and from healthy controls and PMVs isolated by centrifugation. PMVs were subsequently analysed via micro-Bicinchoninic Acid Assay (BCA), NanoSight Tracking Analysis (NTA) (Fig 1A and 1B), and Fluorescence-activated Cell Sorting (FACS) (Fig 1C) to confirm that particles were of the expected size and displayed PMV-associated markers. Total RNA was isolated from purified PMVs and small RNA libraries generated prior to sequencing. Principal component analysis (PCA) distinguished between small RNA sequencing data obtained from T2DM patient derived PMVs and healthy controls (Fig 2A, S1 and S2 Tables). Moreover, subsequent differential gene expression analysis revealed significantly distinct miRNA profiles for T2DM patient-derived PMVs compared with control samples (Fig 2B, S3 Table). Many of the altered miRNAs were down regulated in T2DM (down-regulated = 42) compared up regulated miRNAs (up-regulated = 12). To explore the functions of these altered miRNAs, we performed gene ontology analysis on their experimentally validated mRNA targets (See Methods, S4 Table). We observed that both the up-regulated and down-regulated miRNA-mRNA interactions associate with distinct gene ontologies (Fig 2C, S5 Table). Further, several of the top enriched ontologies were shared between the up and down miRNA-mRNA interactions (Fig 2C, S5 Table), suggesting potential reprogramming within the same core pathways. However, these mRNA targets overlap between the groups (Down: 33% 188 out of 566 and Up: 27% 188 out of 695, S3 Table), which may be related to the fact that a single miRNA can target hundreds of mRNAs. Curation of the miRNAs up-regulated in diabetic PMVs (based on their published functions in cancer [39–42]) revealed an overrepresentation of miRNAs previously reported to act as oncogenes (Fig 2D). Conversely, miRNAs ascribed a tumour-suppressor function were over-represented in genes down-regulated in T2DM-derived PMVs compared with healthy PMV controls (Fig 2D). Collectively, these data show that the miRNA profile of PMVs obtained from T2DM-patients is functionally different to that observed in PMVs obtained from healthy controls and is shifted towards a pro-tumourogenic collection of genes.

### T2DM-derived PMVs demonstrate tropism for TNBC cell uptake

**Fig 3:**
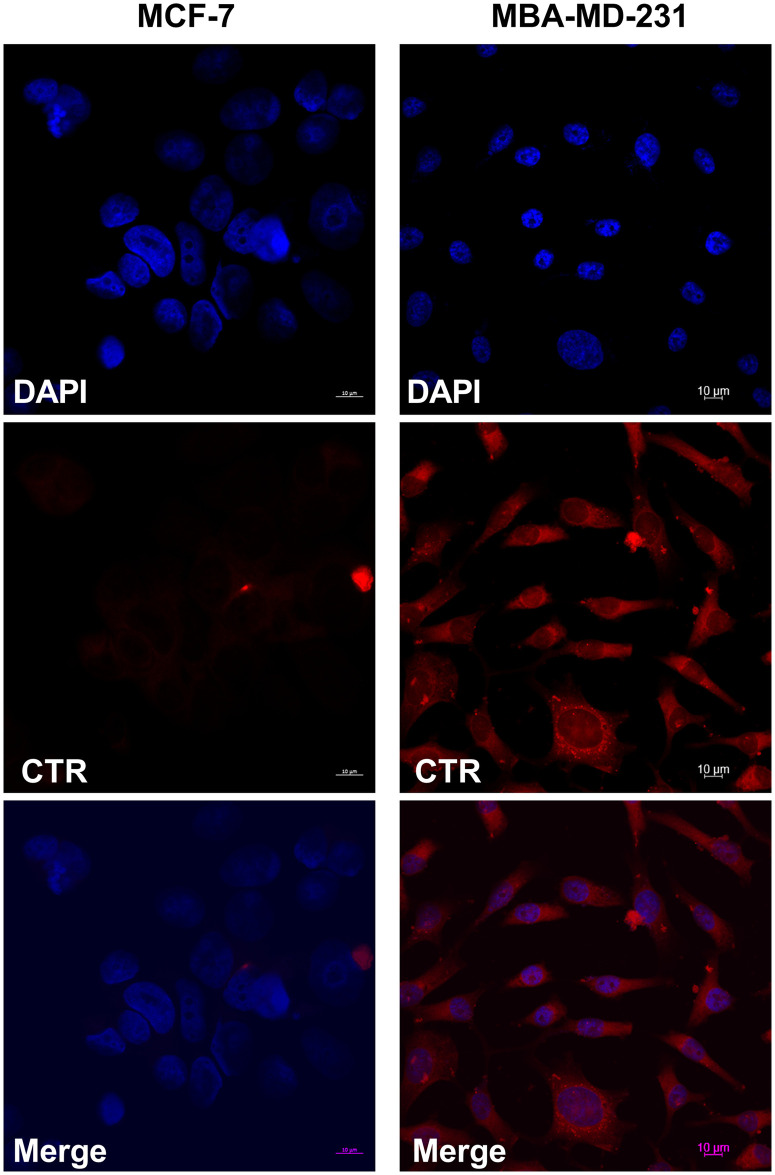
PMVs isolated from poorly controlled diabetic volunteers are internalised by TNBC cells, *in vitro*. PMVs were stained with 1mM CellTracker^™^ Red CMTPX, free-dye removed and labelled PMVs cocultured with MCF-7 or MDA-MB-231 cells for 4h prior to analysis by confocal microscope.

Recently it was reported that PMVs exhibited efficient uptake when co-cultured with the TNBC cell line, MDA-MB-231, but not the luminal ER^+^ Her2^-^ MCF-7 cell line [43]. To determine if PMVs derived from T2DM patient blood exhibited similar preferential uptake, co-culture experiments were carried out with T2DM-derived PMVs labelled with the membrane dye, CellTracker^™^ Red (CTR). Labelled PMVs were cultured with MDA-MB-231 or MCF-7 cells for 30 minutes, prior to fixation and counterstaining with DAPI. As shown in Fig 3, we observed clear uptake of labelled PMVs in MDA-MB-231 cells but only nominal CTR signal in MCF-7 cells, consistent with previously published work^38^. Together these data demonstrate that PMVs from T2DM patients are internalised by TNBC cells, *in vitro*.

### T2DM-derived PMVs potentiate TNBC cell invasion

**Fig 4:**
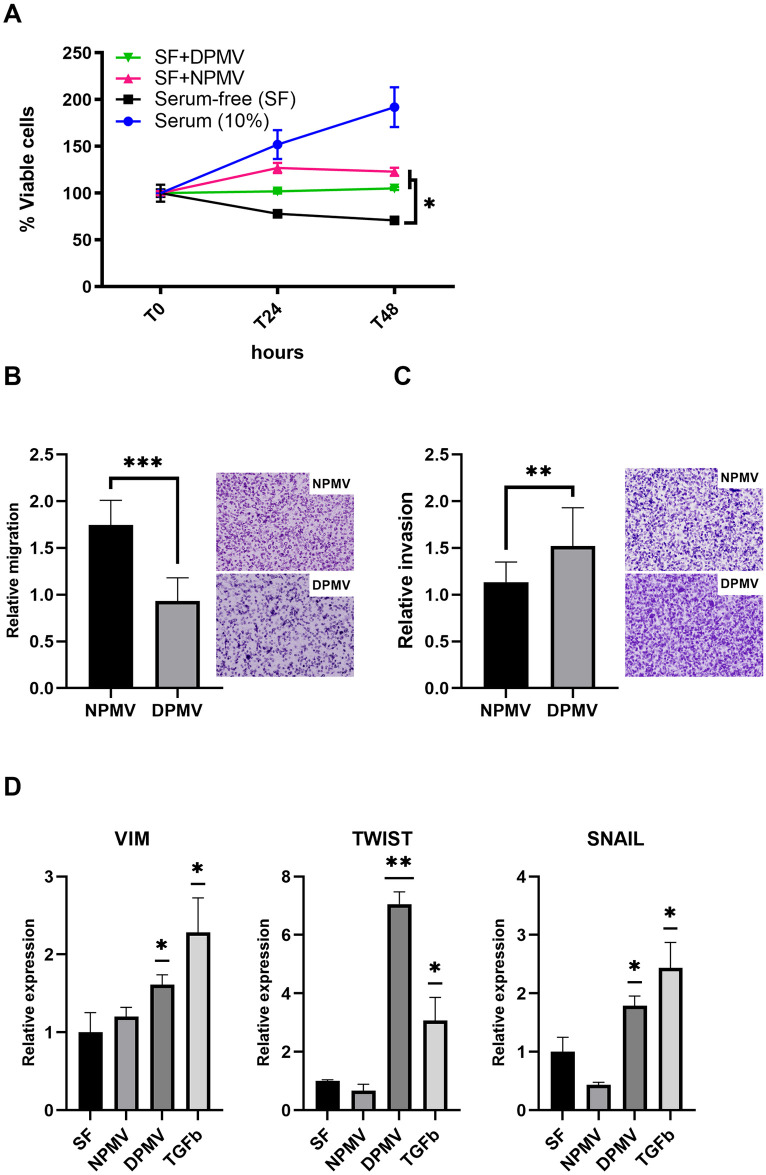
PMVs isolated from poorly controlled diabetic volunteers potentiate TNBC invasion, *in vitro*. (A) MDA-MB-231 cells were cultured for 48h in complete media plus vehicle (PBS), serum-free (SF) media plus vehicle (PBS), SF media with NPMV, or SF media with DPMV and total viable cells assessed via CellTiter-Glo^®^, n = 3. (B and C) MDA-MB-231 cells were cultured in SF media or co-incubated with either healthy control PMVs (NPMV), or diabetic-patient derived PMVs (DPMV) in either uncoated or extra-cellular matrix (ECM)-coated transwell inserts to determine migration (B) and invasion (C), respectively. Migration and invasion rates are relative to cells cultured in SF media, n = 6. (D) MDA-MB-231 cells were cultured in SF media or co-incubated in SF media with either healthy control PMVs (NPMV), diabetic-patient derived PMVs (DPMV), or TGFβ (10ng/ml) for 24h prior to isolation of total RNA and analysis of target gene expression by RT-qPCR, n = 3. * = p>0.05; ** = p<0.01; *** = p<0.001.

PMVs were previously reported to increase MDA-MB-231 cell migration [43]. Given the bias towards oncogenic miRNA cargo in T2DM-derived PMVs we were interested in assessing how T2DM-derived PMVs impact TNBC cells compared with PMVs isolated from healthy volunteers. To investigate this, MDA-MB-231 cells were serum starved prior to co-culture with T2DM-derived PMVs or control PMVs and cell viability, cell migration, and cell invasion assays carried out. As shown in Fig 4A, while co-culture of MDA-MB-231 cells with control and T2DM-derived PMVs led to a nominal increase in total viable cell number 48h post co-incubation compared with control, we observed no significant difference between diabetic and healthy PMVs. Next, we assessed if T2DM-derived PMVs induce cell migration more avidly than control PMVs. MDA-MB-231 cells were cultured in serum-free media prior to seeding in transwell inserts and co-incubation with either vehicle, T2DM-derived PMVs or PMVs isolated from healthy controls. As shown in Fig 4B TNBC cells co-incubated with T2DM-derived PMVs exhibited a significant increase in migration compared with control, however, to our surprise this was not as pronounced as the impact of PMVs from healthy volunteers. Cancer cell invasion is one of the first steps in the metastatic cascade and so we sought to investigate the impact of T2DM-derived PMV co-incubation with TNBC cells on invasion index. MDA-MB-231 cells were cultured in serum-free media prior to seeding in transwell inserts coated with basement membrane matrix (Geltrex^®^) and co-incubated with either vehicle, T2DM-derived PMVs or PMVs isolated from healthy controls. As shown in Fig 4C, PMVs from healthy volunteers did not increase TNBC cell invasion compared with control, however, we observed a significant increase in cell invasion index for TNBC cells co-incubated with PMVs from diabetic patients. Cancer cell invasion is often associated with the expression of drivers of mesenchymal cell state. To determine if T2DM-derived PMVs were increasing the expression of key mesenchymal genes, we analysed the relative expression of *TWIST*, *SNAIL*, and *VIM* (vimentin) via reverse-transcriptase quantitative PCR (RT-qPCR) in TNBC cells co-incubated with T2DM-derived PMVs, PMVs isolated from healthy controls or TGFβ, which served as a positive control for the induction of mesenchymal gene expression. PMVs isolated from healthy controls failed to induce significant increases in mesenchymal genes, however, we observed significant increases in TWIST, SNAIL, and vimentin transcripts levels in cells co-incubated with T2DM-derived PMVs (Fig 4D). Together, these data demonstrate that diabetic PMVs drive a significant increase in TNBC cell invasion, possibly via the induction of EMT-associated genes.

### TNBC cells co-incubated with T2DM-dervied PMV contain increased amount of diabetic PMV-enriched miRNAs

**Fig 5:**
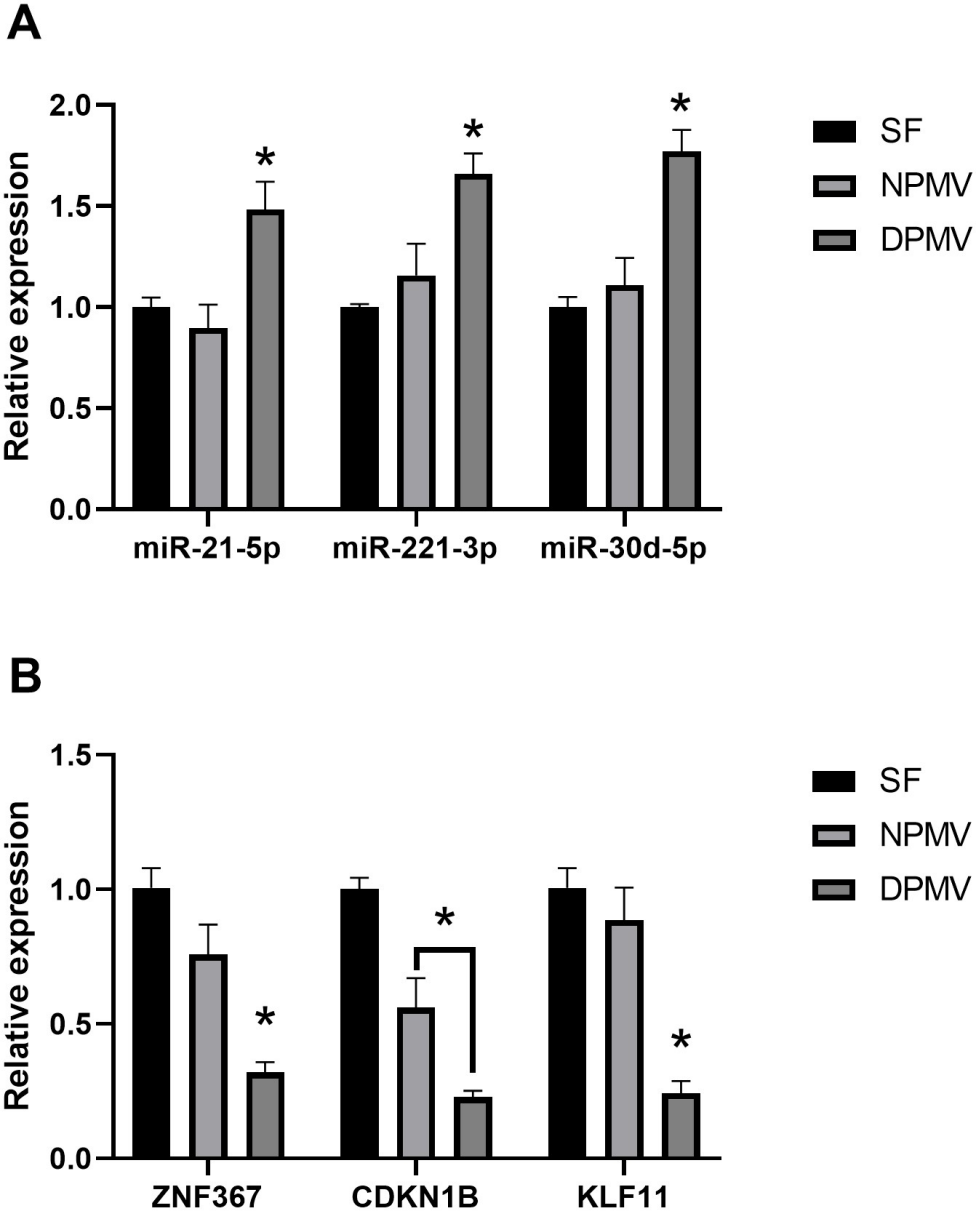
PMVs isolated from poorly controlled diabetic volunteers deliver oncogenic miRNA to TNBC cells, *in vitro*. (A) MDA-MB-231 cells were cultured in the presence of actinomycin D (1 μg/ml) for 24h cultured prior to culturing for a further 24h in serum-free (SF) media, SF media with healthy control PMVs (NPMV), or SF media with diabetic-patient derived PMVs (DPMV). Total RNA was isolated from cells and RT-qPCR performed against target miRNAs, n = 3. (B) Total RNA from (A) was utilised to assess via RT-qPCR the transcript levels of validated cognate targets of each of the miRNA shown in panel A, n = 3. * = p<0.05.

Given the uptake of PMVs by TNBC cells and the enrichment of several protumourigenic miRNAs in our T2DM-derived PMV RNA-seq data, we set out to determine if co-incubation of TNBC cells with T2DM-derived PMVs resulted in increased levels of these miRNAs, when compared with TNBC cells co-incubated with vehicle alone or PMVs isolated from healthy controls. To control for changes in miRNA target levels being driven endogenously via altered expression of cellular pre-miRNAs, TNBC cells were treated with actinomycin D prior to co-incubation with PMVs or control for 48h. Total RNA was isolated, and miRNA transcript levels determined via miRCURY LNA miRNA PCR. As shown in Fig 5A, we observed increased amounts of mir-21-5p, miR-221-3p, and miR-30d-5p in MDA-MB-231 cells co-incubated with diabetic PMVs compared with both control PMVs and vehicle alone. To investigate if these increases corresponded with a functional impact, we assessed the transcript levels of Zinc Finger Protein 367 (ZNF367), Cyclin Dependent Kinase Inhibitor 1B (CDKN1B), and KLF Transcription Factor 11 (KLF11). These three genes have previously been validated, in a breast cancer cell context, to be direct targets of mir-21-5p [44], miR-221-3p [45], and miR-30d-5p [46], respectively. As shown in Fig 5B, we observed a significant decrease in transcript levels in cells treated with PMVs from both healthy and T2DM volunteers, however, this decrease was significantly greater in cells co-incubated with T2DM-derived PMVs, suggesting that elevated levels of mir-21-5p, miR-221-3p, and miR-30d-5p in DPMVs may exert a functional impact on target gene expression in TNBC cells.

## Discussion

Increased platelet activation is common to both T2DM and BC patients, and is a key driver of the hypercoagulable state found in both sets of patients, while also impacting directly on cancer metastasis across a range of neoplasms, including breast cancer [47]. Platelet activation also results in increased release of PMVs and there is compelling evidence that demonstrates a potent correlation between high PMV concentration in the blood and breast cancer progression [48], however, the mechanisms by which PMVs support tumour growth and dissemination remain to be fully elucidated. While a T2DM-induced increase in circulating PMV concentration may contribute to elevated risk of BC, qualitative differences in the miRNA cargo of T2DM patient PMVs may also be influential. This is particularly relevant given the body of research demonstrating that diabetic plasma harbours significantly altered miRNA profiles and the mooted utility of this observation in terms of diagnosis and prognosis of comorbidities [38, 49]. Here we performed small RNA sequencing on PMV total RNA isolated from the blood of poorly controlled T2DM patients and healthy volunteers. We observed a clear differentiation of small RNA transcripts between the two groups via PCA (Fig 2A), suggesting that reported differences in the miRNA profile of platelets in diabetic patients also translates to PMVs being released by these cells. Several studies have reported platelet-specific miRNA expression profiles [29, 50, 51] with a recent study elegantly demonstrating, via subtractive approaches, a panel of platelet-enriched miRNA genes [52]. Analysis of our PMV sequencing data reveals the presence of the widely reported platelet-enriched (PE) miRNAs miR-223-3p and miR-21-5p, moreover, we identified all the PE miRNA genes described by Krammer *et al* [52] in both control and T2DM data, suggesting that platelet-specific miRNA profiles are mirrored in released PMVs. Moreover, DE analysis revealed significant changes across T2DM patient PMV miRNA cargo compared with healthy control PMVs (Fig 2B). In many cases this equated to an alternation in the transcript abundance of PE transcripts, for example miR-223-3p and miR-21-5p were significantly higher in T2DM PMVs, whereas PE miRNA miR-151-5p and miR-148b-3p were significantly decreased.

There is great interest in diagnostic, prognostic, and on-treatment miRNA biomarkers for T2DM, however, findings often lack consensus, likely due to inherent variability between patients and issues with circulating transcript ‘noise’. To assess if observed differences in PMV miRNA levels in our two groups reflected published work reporting on circulating biomarkers for T2DM we cross-referenced our findings with a curated list of circulating T2DM miRNA biomarkers which have appeared and been validated by at least two independent experimental studies [53]. The picture here was mixed with some of our differential targets (miR-24-3p↓, miR-146a-5p↑, miR-30d-5p↑) mirroring abundance changes reported in diabetic patient plasma, however, both miR-126-3p and miR-223-3p are decreased in T2DM patient plasma but increased in our T2DM PMVs. One explanation for this could be the small sample size used in this study, but it is also likely that changes to circulating miRNA levels in diabetic patients arise via several routes and that the input from platelet-derived miRNA is just one of many contributing factors.

The focus of this study was the impact of diabetic PMVs on BC. Inspection of our T2DM PMV miRNA data in terms of changes to genes that have been shown to contribute to pro- and anti-tumourogenic progression in breast cancer [39–42], revealed a skewed pattern of changes in T2DM PMVs that favoured increased miRNA transcript levels for genes that have been previously reported to function in a pro-tumourogenic capacity in breast cancer (Fig 2D), including miR21-5p, miR-221-3p, and miR-30d-5p [46, 54–57]. Understanding how observed DE in miRNA genes between T2DM and control PMV relates to both the diabetic state of the donor and wider biology, specifically breast cancer cell growth and progression is to an extent a Gordian knot. To begin unravelling this, one approach is to focus on those differentially expressed PMV miRNAs upregulated in T2DM plasma that also have reported roles in BC progression. Two genes that meet these criteria in our data are miR-21-5p and miR-30d-5p, indeed a single study reported that miR-30d-5 expression correlates with both insulin resistance and BC development, however, it is important to note that this was generally in the context of ER+ BC and so if this also applies to a TNBC context is unclear [58]. To investigate the impact of increased pro-tumourogenic miRNA transcripts in T2DM PMVs on BC cells a series of *in vitro* co-incubation experiments were performed. Previously, PMVs have been reported to exhibit efficient internalisation by the TNBC cell line, MDA-MB-231, and in contrast poor uptake by the ER+ BC cell line, MCF-7 [43], an observation recently confirmed via a second study [59]. We observed similar results using T2DM PMVs, with only nominal uptake in MCF-7 cells compared with extensive uptake in MDA-MB-231 cells (Fig 3). The mechanisms governing PMV internalisation are poorly understood. There is some evidence that in endothelial cells uptake occurs via active endocytosis [60], and that in breast cancer cells PMV binding is integrin αIIbβ3-independent, however, αIIbβ3 antagonists reported to be ineffective at blocking PMV uptake into MDA-MB-231 cells [43]. Limited amounts of clinical sample prevented more widespread analysis of PMV uptake across additional BC cells lines in this study, however, more research is needed, both in terms of PMV cell surface proteins and their contribution to target cell tropism and to the precise mechanisms governing uptake and if these are uniform across different target cell types.

Co-incubation of MDA-MB-231 cells with PMVs resulted in increased cell growth and migration, as previously reported [43], however, there was no significant T2DM-specific impact on cell growth and surprisingly while migration was increased in MBA-MD-231 cells co-incubated with T2DM-derived PMVs this was at a significantly lower level than PMVs isolated from healthy controls (Fig 4A and 4B). In contrast, co-incubation with T2DM- derived PMVs, resulted in a significant increase in MBA-MD-231 cell invasion and expression of EMT-associated genes compared with PMV controls (Fig 4C and 4D). As PMV concentration (mg/mL) used in *in vitro* assays was the same for control and diabetic samples, the invasion-specific effect of T2DM PMVs on TNBC cells is presumably due to qualitative differences in these PMVs in terms of either their uptake efficiency, the cargo they deliver to cells, or both. We observed no obvious difference in MBA-MD-231 PMV uptake between diabetic and control, however, it is important to note that the method employed here does not facilitate true quantitative analysis of PMV uptake and therefore we cannot exclude the possibility that the phenotypic and biochemical differences we observed in MBA-MD-231 cells are due to a general increase in diabetic PMV internalisation, and that our analysis of EMT gene expression was limited to analysis of RNA transcript levels. Interestingly, we did observe an increase T2DM-enriched PMV miRNA in MBA-MD-231 cells co-incubated with diabetic PMVs compared with control, suggesting that the increased abundance of these miRNAs in diabetic PMVs results in elevated levels of these miRNAs in target cells. Importantly, this increase was observed in cells pre-treated with the RNA polymerase II inhibitor, actinomycin D, suggesting that elevated levels were due to transfer of miRNA from PMVs, rather than indirect effects of PMV co-incubation on miRNA transcription (Fig 5A). Furthermore, quantitative analysis of reported cognate mRNA targets of miRNAs elevated in diabetic PMVs revealed a significant decrease in transcript abundance compared with the same targets in MBA-MD-231 cells co-incubated with control PMVs, suggesting that the increased levels of some miRNA in T2DM-derived PMVs manifested a tangible and significant impact on the levels of target messenger RNA in BC cells (Fig 5B). Several studies have demonstrated that high levels of miR-21-5p positively correlate with poor outcome and increased invasive index in TNBC [61–63], with one study reporting that increased levels of miR-21-5p in serum is associated with TNBC metastasis and prognosis [63]. While we observed changes in miRNA levels of TNBC cells co-incubated with PMVs, it is likely that PMV protein cargo and PMV-associated receptors also exert an influence or target cells, further investigation is required to better understand how these biomolecules differ between healthy and diabetic PMVs.

A limitation of this study is that our phenotypic assessment of patient derived PMVs occurs only through *in vitro* based assays. Conceptually, the likely impact of a differential PMV miRNA signature for T2DM patients who develop BC is via altered concentrations and cargo of circulating PMVs in the blood. PMVs represent the overwhelming majority (up to 90%) of all cell-derived vesicles in blood [17, 64] and as such it is conceivable that changes to platelet biology and by extension the microvesicles that they shed in disease like T2DM might have a measurable impact on systemic biology, including development and progression of cancer. Moreover, evidence demonstrating that PMVs increase endothelial cell permeability suggest a manner by which these vesicles might access tumour cells [65]. Clearly, clinical and *in vivo* studies are sorely needed, however, attempting to disentangle PMV-induced effects from circulating extracellular vesicles is an ongoing challenge in the field [66]. With regards to the T2DM PMV miRNA profile described in this study, while we observed a pro-tumourogenic consensus across increased miRNAs, it is important that we do not discount the fact that numerous changes to the DE of miRNAs in T2DM-derived PMV compared with healthy control are represented by significant decreases in miRNAs reported to function in an anti-tumourogenic capacity (Fig 2D). It seems possible that a dramatic decrease in a PMV-associated circulating pool of anti-tumourogenic miRNA, due to T2DM, may propagate an environment that is more receptive of both tumourigenesis and cancer metastasis. This model is also consistent with studies that have reported PMVs to function in a predominantly protective manner [67]. Interestingly, decreased miRNAs in our T2DM-derived PMV data include miR-199a-5p and miR-221-5p, which have previously been linked to tumour-suppressive roles in TNBC [68, 69].

**Fig 6:**
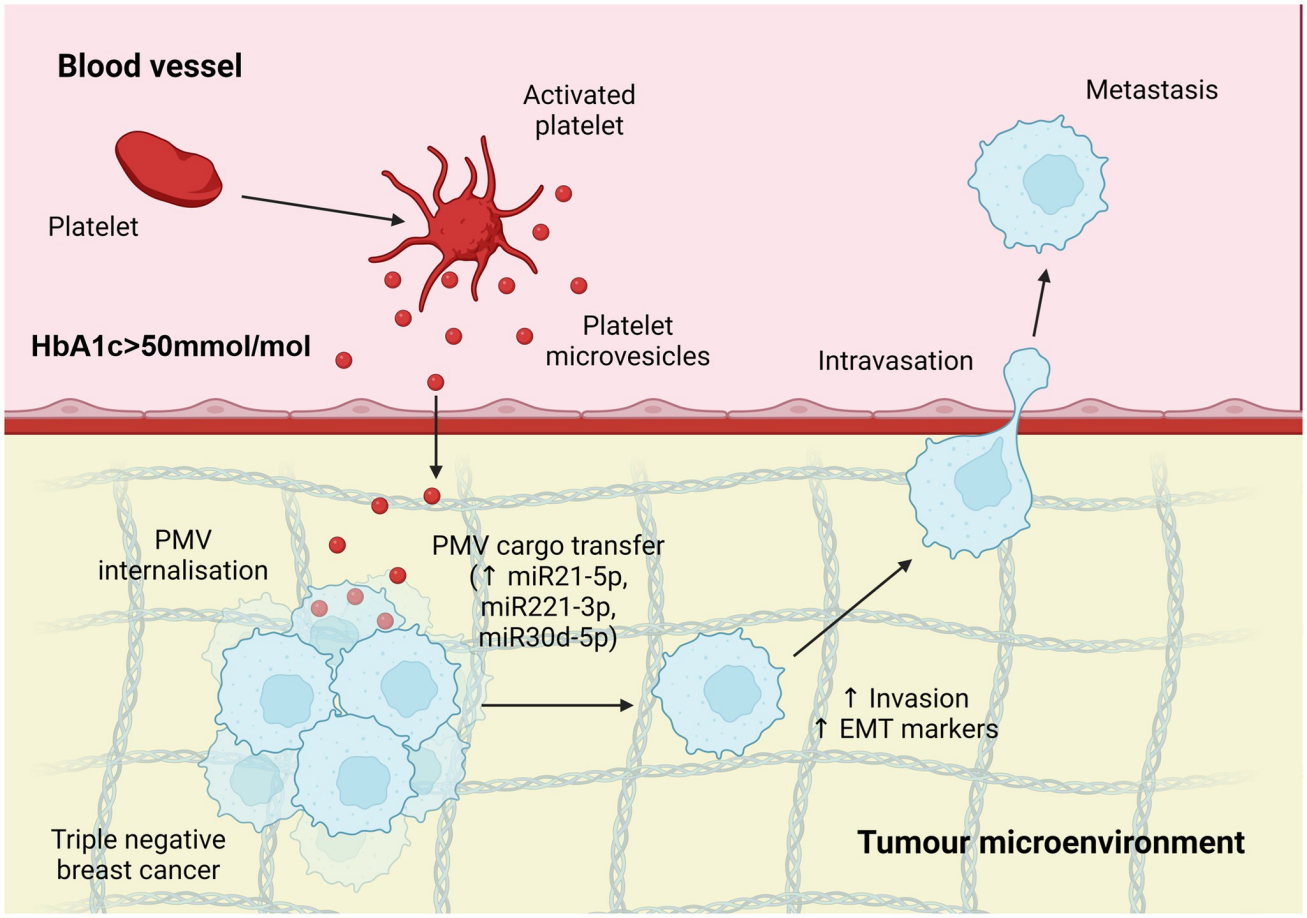
Model for how DPMV might impact on TNBC progression. Circulating PMVs harbour an altered and pro-oncogenic miRNA profile in T2DM patients (indicated by HbA1c>50mmol/mol) that impact on TNBC invasion, *in vitro*, and might similarly drive tumourigenesis in diabetic patients with breast cancer.

In summary, we have shown that PMVs isolated from poorly controlled T2DM patients harbour a distinct onco-miR-enriched miRNA profile, are taken up by TNBC cells and potentiate cell invasion (Fig 6). In addition, with the utility of extracellular vesicle biomarker signatures for BC becoming increasingly clear [70–72], future studies on a larger patient cohort will be essential to determine if a suitable miRNA signature can be identified within diabetic PMVs that might be used to identify diabetic patients at increased risk of developing breast cancer or indeed diabetic breast cancer patients at risk of developing metastatic disease.

## Methods

### PMV isolation and analysis

Ethical approval was obtained prior to the start of this study (IRAS project ID 235632). The recruitment period for the study was 31^st^ May 2018 to 31^st^ May 2020. Written informed consent, witnessed, and documented by a research nurse (JT), was obtained from participants prior to the collection of samples. Samples were stored in a fully anonymised form. PMV isolation was carried out as previously described [23]. Briefly, human blood was taken by venepuncture from healthy adult volunteers and T2DM patients with HbA1C>50 mmol/mol, anonymised demographic data for consented patients can be seen in S6 Table. Blood was added to acid citrate dextrose (ACD; 29.9mM sodium citrate, 113.8mM glucose, 72.6mM sodium chloride and 2.9mM citric acid, pH 6.4) and platelet-rich plasma (PRP) was obtained by centrifugation of whole blood at 200g at 20°C, 20 minutes. Washed platelets were isolated from the PRP by centrifugation at 800g at 20°C, 12 minutes in the presence of prostaglandin E1 (PGE1; 50 ng/ml). Platelets were resuspended in Tyrode’s buffer (150mM NaCl, 5mM HEPES, 0.55mM NaH_2_PO_4_, 7mM NaHCO_3_, 2.7mM KCl, 0.5mM MgCl_2_, 5.6mM glucose). PMV were isolated as reported [23] and suspensions activated with thrombin (0.1 U/ml) for 60 minutes. Platelet activation was stopped by EDTA (20 mM) and platelets pelleted by centrifugation (3200g, 10 minutes). PMVs were isolated by centrifugation at 20,000g for 90 minutes at 18°C, then resuspended in Tyrode’s buffer. Protein content was measured using micro-BCA assay (Thermofisher Scientific, UK), and 100mg/ml of pooled PMV suspension used in all assays. PMVs were diluted 100-fold in PBS and analysed by flow cytometry as previously reported [21, 31] for phosphatidylserine exposure detected by Fluorescein (FITC)-Annexin-V (BD Biosciences) binding or platelet specific marker CD41a expression detected with APC-conjugated anti human CD41a antibody or APC-conjugated mouse IgG isotope control (BD Biosciences). Following incubation for 30 minutes, samples were diluted 5-fold in PBS and FACS analysis was completed using the BD Accuri^™^ C6 Flow Cytometer (BD Biosciences, UK), making sure to QC instrument before each use using the BD CS&T RUO Beads (BD Biosciences, UK). For tracking cellular uptake PMVs were stained with 1mM CellTracker^™^ Red CMTPX Dye (ThermoFisher, UK) for 30 minutes at 37°C and incorporated dye removed via centrifugation in Exosome Spin Columns (ThermoFisher, UK) prior to co-incubation with cells for 4 hours, followed by fixation in 4% paraformaldehyde for 10 minutes, staining with DAPI and visualization via LSM 880 with Airyscan (Zeiss).

### Cell culture

Breast cancer cell lines were obtained from European Collection of Authenticated Cell Cultures and certified mycoplasma-free. MDA-MB-231 and MCF-7 cells were maintained in Dulbecco’s Modified Eagle Medium (DMEM; ThermoFisher, UK), supplemented with 10% foetal bovine serum (FBS), 100 U/ml of penicillin and 100 μg/ml of streptomycin. All cells were cultured at 37°C and 5% CO_2_. LNA inhibitors were transfected into cells using HiPerfect (QIAgen, UK), as previously described [23]. To assess PMV transfer of miRNA, MDA-MB-231 cells were treated with actinomycin D (1mg/mL), as previously described [73]. For RT-qPCR analysis of EMT markers, cells were co-incubated with 10ng/ml of TGFβ as a positive control for induction of mesenchymal gene expression.

### Viable cell growth

Cells were seeded at a density of 1x10^4^ cells/well in white-walled 96-well plates (ThermoFisher, UK). After 24 hours, media was replaced with 100μl of serum-free media (SFM) and cells incubated at 37°C for a further 24, 48 and 72 hours in the presence of either vehicle alone (PBS), NPMV, DPMV or 10% FBS. Viable cell growth was assessed at each time point using CellTiter-Glo^®^ Luminescent Cell Viability Assay (Promega, UK) and luminescence recorded 10 minutes after reagent addition using the GloMax^®^ Explorer system (Promega, UK).

### Transwell assays

Cells were grown for at least 24 hours in serum media, then serum starved for 24 hours prior to detachment and resuspension in the indicated media at a concentration of 1x10^5^ cell/ml. Depending on the experiment, 1ml of serum-free or complete media was added to the lower chambers of a 24 well plate and 500μL of the cell suspension added to an 8μm tissue culture (TC)-insert (Sarstedt, UK). For invasion assays, TC-inserts were coated with 50μL Geltrex^™^ LDEV-Free Reduced Growth Factor Basement Membrane Matrix (Gibco) and incubated at 37°C for 1 hour. To assess migration and invasion index, cells were incubated for 24 hours with vehicle or PMVs at 37°C and a cotton bud used to gently remove any non-migrated cells from the apical side of the transwell insert membrane. Prior to being fixed with ice cold 70% ethanol for 30 minutes. Insert membranes were stained in 0.2% crystal violet (Fisher Science, UK) for 10 minutes, then mounted onto slides. 5 random high-power images were captured (EVOS XL Core Cell Imaging System, 4x objective) and the number of migrated cells calculated, relative to serum-free control.

### RNA extraction and RT-qPCR

Total RNA was extracted from PMVs, or PMV treated cells using the Aurum total RNA Mini kit (BIO-RAD, UK) according to the manufacturer’s instructions. To assess PMV transfer of miRNA, cells were treated with actinomycin D (1mg/mL), as previously described [73]. For RT-qPCR analysis of EMT markers, cells were co-incubated with 10ng/ml of TGFβ as a positive control. 500ng of total RNA was used to generate cDNA using SsoAdvanced SYBR master mix (Bio-Rad, UK), and RT-qPCR analysis carried out using the CFX96 system (Bio-Rad, UK). Micro-RNA expression was analysed using miRCURY LNA miRNA PCR assays (Qiagen, UK) and data normalised against UniSp6 spike RNA. A list of oligonucleotides used during this study can be found in S7 Table.

### RNA sequencing analysis

Small RNA-Seq libraries were prepared using NEBNext^®^ Ultra^™^ DNA Library Prep Kit for Illumina^®^ (NEB, UK) and analysed via NextSeq 500 (Illumina, USA) and data deposited at GEO (TBC). Raw reads were subjected to adaptor trimming (Illumina sequencing adapters) using Cutadapt v2.5 [74]. Trimmed reads were aligned to the GRCh38/hg38 assembly of the human genome using Bowtie2 v2.3.5.1 [75]. HTSeq-count v0.11.1 [76] was used to quantify the miRNA abundance based on the human miRNA annotation (miRBase Release 22.1) with parameters (-s no -a 10 -m union—nonunique none). Expression levels were normalised by “counts per million” (CPM). Differential expression (DE) analyses between diabetic and non-diabetic samples were performed using the edgeR R package [77]. The DE microRNAs were defined at adjusted p-value < 0.05.

### Gene ontology analysis

The mRNA targets of the DE miRNAs were extracted from the miRTarBase database [78]. To reduce the rate of false-positive enrichments, only experimentally validated miRNA-mRNA pairs were extracted for further analysis. Biological processes associated with the mRNA targets were determined using R ClusterProfiler and the human Bioconductor annotation database (org.Hs.eg.db) to compare the enriched biological processes between diabetic and non-diabetic targets. All enrichment analyses were performed using p-value and q-value <0.01 cut-off with reduced redundancies by semantic similarity analysis [79].

### Statistical analysis

Except stated otherwise, graphical data shown represent mean ± standard deviation of mean (SD) from at least three independent experiments. Differences between means was analysed by Student’s t-test, verified by Bonferroni, Mann Whitney Wilcoxon test, or ANOVA. Statistics were considered significant at p<0.05.

## Supporting information

S1 Table miRNA transcript count. Raw count of miRNA transcript across all samples.
(XLSX)

S2 Table Normalised data. Le CPM normalised expression levels of miRNA transcripts across all samples.
(XLSX)

S3 Table Differential expression of miRNA. Differential expression results for miRNAs passing minimum expression threshold (1 CPM in 3 samples).
(XLSX)

S4 Table miRNA targets. Functionally validated miRNA-mRNA interactions for the differentially expressed miRNA in S3 Table found miRTarBase.
(XLSX)

S5 Table Gene ontology analysis. Complete gene ontology analysis of molecular functions associated the target mRNA between each group.
(XLSX)

S6 Table T2DM patient data. Anonymised data for T2DM patients recruited to the study.
(XLSX)

S7 Table Oligonucleotides. Details of oligonucleotides used in this study.
(XLSX)
